# Homocoupling of aryl halides in flow: Space integration of lithiation and FeCl_3_ promoted homocoupling

**DOI:** 10.3762/bjoc.7.122

**Published:** 2011-08-02

**Authors:** Aiichiro Nagaki, Yuki Uesugi, Yutaka Tomida, Jun-ichi Yoshida

**Affiliations:** 1 Department of Synthetic Chemistry and Biological Chemistry, Graduate School of Engineering, Kyoto University, Kyotodaigakukatsura, Nishikyo-ku, Kyoto, 615-8510, Japan

**Keywords:** homocoupling, iron salts, microreactor, organolithiums

## Abstract

The use of FeCl_3_ resulted in a fast homocoupling of aryllithiums, and this enabled its integration with the halogen–lithium exchange reaction of aryl halides in a flow microreactor. This system allows the homocoupling of two aryl halides bearing electrophilic functional groups, such as CN and NO_2_, in under a minute.

## Introduction

Biaryl structures often occur in various organic compounds including natural products, bioactive compounds, functional polymers, ligands in catalysts and theoretically interesting molecules, and the oxidative homocoupling of arylmetals is one of the most useful methods for the construction of biaryl frameworks [1]. Stoichiometric amounts of transition metal salts such as TiCl_4_ [2], TlCl [3], VO(OEt)Cl_2_ [4], CoCl_2_ [5], CuCl_2_ [6] and Pd(OAc)_2_ [7] have been used for homocoupling of arylmetals. In some cases catalytic processes in the presence of a reoxidant, such as oxygen or other organic oxidants, are effective. Recently, iron salts have been also used because of their low costs and lack of toxicity [8–18]. For example, Hayashi et al. reported the iron-catalyzed oxidative homocoupling of Grignard reagents, using 1,2-dihalogenoethanes as an oxidant [19]. Cahiez et al. have also reported the FeCl_3_-catalyzed homocoupling reaction of Grignard reagents bearing functional groups, using atmospheric oxygen [20]. The use of aryllithium compounds instead of Grignard reagents is very interesting, because they are easily generated by halogen–lithium exchange under homogeneous conditions, thus enabling the generation in a flow. However, to the best of our knowledge, oxidative homocoupling of aryllithiums using iron salts has not been reported so far. One of the major reasons for this seems to be the instability of aryllithiums, especially of those bearing electrophilic functional groups such as cyano and nitro groups [21], making the subsequent homocoupling difficult or impossible.

Recently, we have reported that flow microreactor systems [22–85] are quite effective for the generation and reaction of highly reactive organolithiums such as functionalized aryllithiums, oxiranyllithums, aziridinyllithiums, and allenyllithiums [86–98]. Herein we report that integration [99–100] of the generation of aryllithiums, especially those bearing electrophilic functional groups, by halogen–lithium exchange and FeCl_3_ promoted homocoupling has been effectively accomplished in an integrated flow microreactor system.

## Results and Discussion

**Scheme 1:**
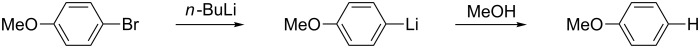
Halogen–lithium exchange of *p*-bromoanisole followed by reaction with methanol.

**Figure 1:**
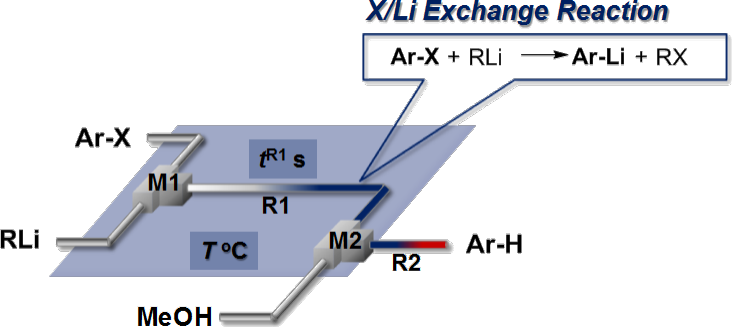
Flow microreactor system for halogen–lithium exchange of aryl halide followed by reaction with methanol. T-shaped micromixer: **M1** (inner diameter: 250 μm), and **M2** (inner diameter: 500 μm), microtube reactor: **R1** and **R2** (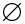 = 1000 μm, length = 50 cm), a solution of aryl halides: 0.10 M in THF (6.0 mL/min), a solution of lithium reagent: 0.40 M or 0.42 M in hexane (*n*-BuLi) or Et_2_O (PhLi) (1.5 mL/min), a solution of methanol: Neat (3.0 mL/min).

**Figure 2:**
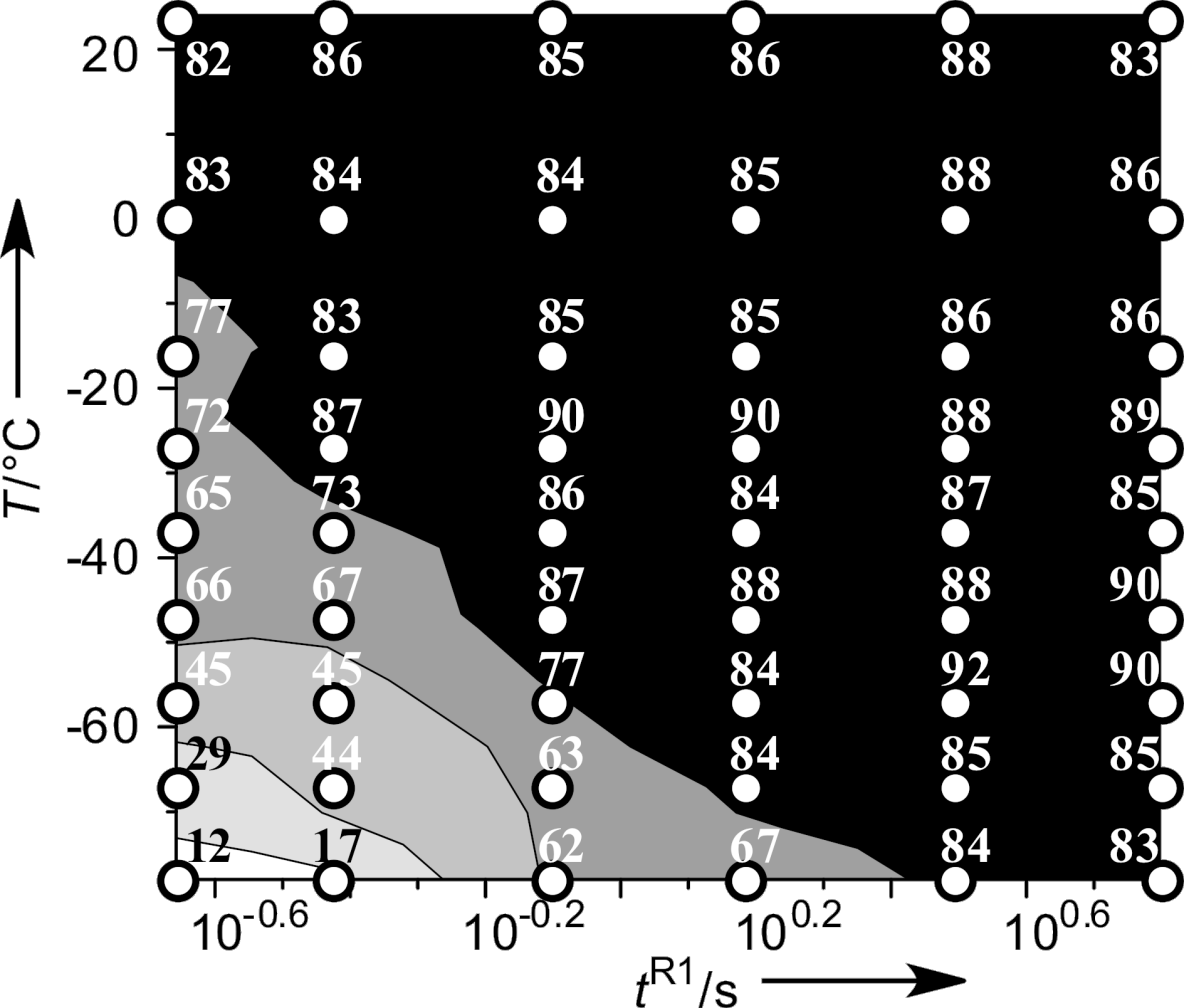
Effects of the temperature (*T*) and the residence time in **R1** (*t*^R1^) on the yield of anisole in the Br–Li exchange reaction of *p*-bromoanisole followed by reaction with methanol in the flow microreactor system. The contour plot with scatter overlay shows the yields of anisole (%), which are indicated by small circles.

First, we focused on the generation of *p*-methoxyphenyllithium from *p-*bromoanisole (Scheme 1). A flow microreactor system, consisting of two T-shaped micromixers (**M1** and **M2**) and two microtube reactors (**R1** and **R2**) shown in Figure 1, was used. A solution of *p-*bromoanisole (Ar–X) (0.10 M in THF, flow rate: 6.0 mL/min) and a solution of *n*-butyllithium (0.40 M in hexane, flow rate: 1.5 mL/min) were introduced to **M1** (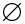 = 250 μm) by syringe pumps. The resulting mixture was passed through **R1** to conduct the bromine–lithium exchange reaction. Methanol (neat, flow rate: 3.0 mL/min) was added in **M2** (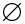 = 500 μm) and the mixture was passed through **R2** (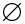 = 1000 μm, L = 50 cm) to protonate *p*-methoxyphenyllithium. The reactions were carried out with varying residence time in **R1** (*t*^R1^: 0.2–6.3 s) and varying temperature (*T*: −78 to 24 °C). The temperature (*T*) was controlled by adjusting the bath temperature. The residence time (*t*^R1^) was adjusted by changing the inner diameter and the length in the microtube reactor **R1** with a fixed flow rate. After a steady state was reached, the product solution was collected for 30 s. As shown in Figure 2, the yield of the protonated product, anisole, depends on both *T* and *t*^R1^. The reaction at low temperatures (*T* < −48 °C) with short residence times (*t*^R1^ < 0.79 s) resulted in very low yields, because the Br–Li exchange reaction was not complete. The increase in *T* and *t*^R1^ caused an increase in the yield, and high yields (>85%) were obtained through the appropriate choice of *T* and *t*^R1^.

**Scheme 2:**
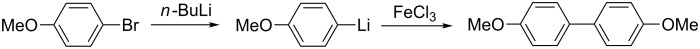
Halogen-lithium exchange of *p*-bromoanisole followed by oxidative homocoupling with FeCl_3_.

**Figure 3:**
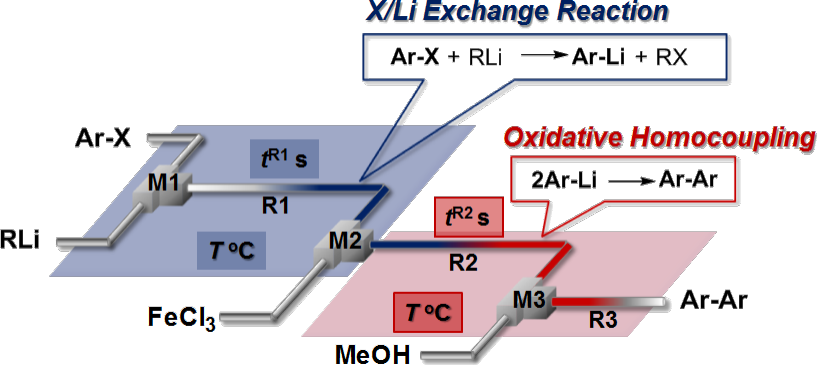
Integrated flow microreactor system for oxidative homocoupling reaction of aryllithium with FeCl_3_. T-shaped micromixer: **M1** (inner diameter: 250 μm), **M2** (inner diameter: 500 μm), and **M3** (inner diameter: 500 μm), microtube reactor: **R1**, **R2** and **R3** (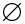 = 1000 μm, length = 50 cm), a solution of aryl halides: 0.10 M in THF (6.0 mL/min), a solution of lithium reagent: 0.42 M in hexane (*n*-BuLi) or Et_2_O (PhLi) (1.5 mL/min), a solution of FeCl_3_: 0.10 M in THF (6 mL/min), a solution of methanol: Neat (1.5 mL/min).

**Figure 4:**
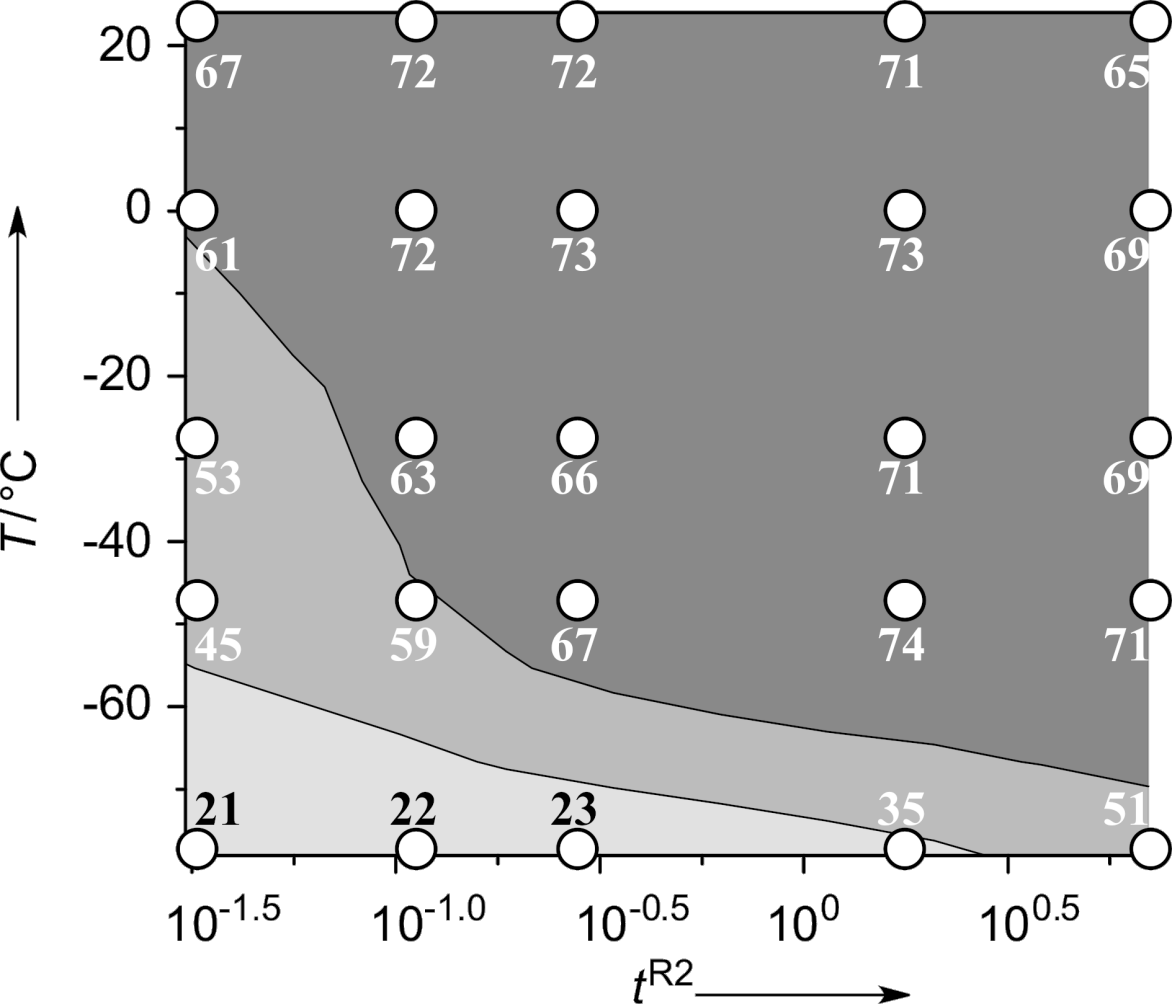
Effects of the temperature (*T*) and the residence time in **R2** (*t*^R2^) on the yield of 4,4'-dimethoxybiphenyl in the oxidative homocoupling of *p*-methoxyphenyllithium with FeCl_3_ in the integrated flow microreactor system. Contour plot with scatter overlay of the yields of 4,4'-dimethoxybiphenyl (%), which are indicated by small circles.

Next, we examined the integration of the halogen–lithium exchange reaction with FeCl_3_ promoted homocoupling (Scheme 2). Integrated flow microreactor systems consisting of three micromixers (**M1**, **M2**, and **M3**) and three microtube reactors (**R1**, **R2**, and **R3**) were used, as shown in Figure 3. A solution of *p*-bromoanisole (Ar–X) (0.10 M in THF, flow rate: 6.0 mL/min) and a solution of *n*-butyllithium (0.42 M in hexane, flow rate: 1.5 mL/min) were introduced to **M1** (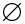 = 250 μm) by syringe pumps. The resulting mixture was passed through **R1** (*t*^R1^ = 13 s (−78 °C), *t*^R1^ = 13 s (−48 °C), *t*^R1^ = 3.1 s (−28 °C), *t*^R1^ = 3.1 s (0 °C), *t*^R1^ = 3.1 s (24 °C)) at the corresponding temperatures and was mixed with a solution of FeCl_3_ (0.10 M in THF, flow rate: 6.0 mL/min) in **M2** (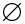 = 500 μm). The resulting mixture was passed through **R2** and was then mixed with methanol (neat, flow rate: 1.5 mL/min) in **M3** (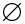 = 500 μm) to protonate the unchanged *p*-methoxyphenyllithium. The resulting solution was passed through **R3** (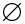 = 1000 μm, L = 50 cm). The temperature (*T*) was controlled by adjusting the bath temperature, and the residence time in **R2** (*t*^R2^) by changing the inner diameter and the length in **R2** with the fixed flow rate. After a steady state was reached, the product solution was collected for 30 s. The results obtained from varying *t*^R2^ and *T* are summarized in Figure 4, in which the yield of 4,4'-dimethoxybiphenyl is plotted against *T* and *t*^R2^ as a contour map with scattered overlay (see Supporting Information File 1 for details). The yield depends on both *T* and *t*^R2^. At −78 °C, the yield increased with *t*^R2^ because of the progress of the homocoupling. At 0 °C, the homocoupling product was obtained in reasonable yields for a wide range of *t*^R2^. The productivity of the present system is acceptable for large scale laboratory synthesis (6.2 g/h). It is noteworthy that the integrated reactions were complete within the overall residence time of 14.7 s, even at low temperatures such as −48 °C. Thus, we envisaged that the reaction could also be applied to less stable aryllithium compounds that decompose very quickly.

One of the major benefits of flow microreactor synthesis is the ability to use highly unstable reactive intermediates. Such intermediates can be rapidly generated and transferred to another location to be used in a subsequent reaction before they decompose. We have already reported the generation and reactions of unstable aryllithium species such as *o*-bromophenyllithiums, and aryllithiums bearing alkoxycarbonyl, cyano, nitro, and ketone carbonyl groups [86–87,93,95–96,98], which are difficult to use in conventional macro batch reactors. As shown in Table 1, reactions of aryllithiums bearing cyano and nitro groups proceeded successfully to give the corresponding homocoupling products, where in contrast it is very difficult to achieve such reactions using conventional batch reactors. A mechanism involving transmetalation of the aryl group from lithium to iron followed by reductive elimination of the homo-coupling product seems to be plausible, while a similar mechanism is proposed for homo-coupling of organomagnesium compounds with FeCl_3_ [19–20]. The regiospecificity of the coupling is consistent with this mechanism. Radical coupling seems to be less likely.

**Table 1:** Homocoupling of aryl halides using the integrated flow microreactor system.

Ar–X	*T* (°C)	*t*^R1^ (s)	Ar–Ar	Yield (%)

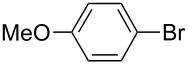	24	3.100	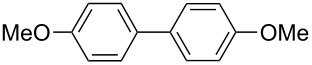	72
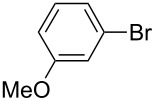	24	3.100	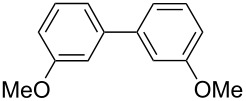	69
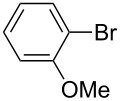	0	3.100	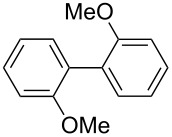	76
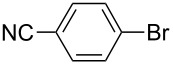	−28	0.055	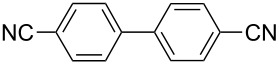	75
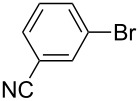	0	0.055	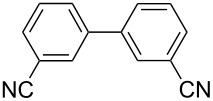	66
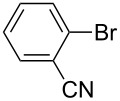	24	0.055	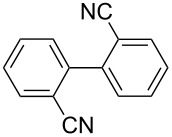	76
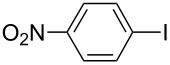	−48	0.014	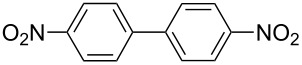	53^a^
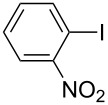	−48	0.014	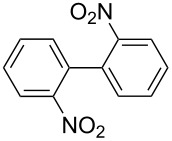	63^a^

^a^PhLi instead of *n*-BuLi was used as lithiating reagent.

## Conclusion

In conclusion, we found that the use of FeCl_3_ results in fast oxidative homocoupling of aryllithiums, which enables its integration with the halogen–lithium exchange of aryl halides. Various aryl halides, including those bearing electrophilic functional groups, can be used for this transformation in the integrated flow microreactor system. Hence, the method greatly enhances the synthetic utility of aryllithium compounds and adds a new dimension to the chemistry of coupling reactions.

## Supporting Information

Supporting Information features experimental procedures and full spectroscopic data for all new compounds.

File 1 Experimental details.
