# Supplementary material for: Homocoupling of aryl halides in flow: Space integration of lithiation and FeCl3 promoted homocoupling
Source: Beilstein J Org Chem. 2011 Aug 2;7:1064–9. doi: 10.3762/bjoc.7.122 (PMC3170206; doi:10.3762/bjoc.7.122)
Supplement: File 1 — Experimental details. [file Beilstein_J_Org_Chem-07-1064-s001.pdf]

Supporting Information

for

Homocoupling of aryl halides in flow: Space integration of lithiation and $FeCl_3$ promoted homocoupling

Aiichiro Nagaki, Yuki Uesugi, Yutaka Tomida, and Jun-ichi Yoshida*

Address: Department of Synthetic Chemistry and Biological Chemistry, Graduate School of Engineering, Kyoto University, Kyotodaigakukatsura, Nishikyo-ku, Kyoto, 615-8510, Japan

Email: Jun-ichi Yoshida* - yoshida@sbchem.kyoto-u.ac.jp

* Corresponding author

Experimental details

General

GC analysis was performed on a SHIMADZU GC-2014 gas chromatograph equipped with a flame ionization detector using a fused silica capillary column (column, CBP1; 0.25 mm x 25 m; initial oven temperature, 50 ^{o}C; rate of temperature increase, 10 ^{o}C/min). ^{1}H and ^{13}C NMR spectra were recorded on Varian MERCURYplus-400 (^{1}H 400 MHz, ^{13}C 100 MHz) spectrometer with Me_4Si or $CDCl_3$ as a standard in $CDCl_3$ unless otherwise noted. THF was purchased from Kanto Chemical Co., Inc. as a dry solvent and used without further purification. Hexane was purchased from Wako, distilled before use, and stored over molecular sieves (4 Å). *p*-Bromoanisole, *m*-bromoanisole, *o*-bromoanisole, *p*-bromobenzonitrile, *m*-bromobenzonitrile, *o*-bromobenzonitrile, *p*-iodonitrobenzene, *o*-iodonitrobenzene, methanol, iron (III) chloride and organolithium reagents were commercially available. Stainless steel (SUS304) T-shaped micromixer with inner diameter of 250 and 500 μm was manufactured by Sanko Seiki Co., Inc. Stainless steel (SUS316) microtube reactors with inner diameter of 250, 500 and 1000 μm were purchased from GL Sciences and were cut into appropriate lengths (3.5, 6, 12.5, 25, 50, 100, 200 and 400 cm). The micromixers and the microtube reactors were connected with stainless steel fittings (GL Sciences, 1/16 OUW). The flow microreactor system was dipped in a cooling bath to control the temperature. Harvard Model 11, equipped with gastight syringes purchased from SGE was used to introduce the solution into a flow microreactor system. After a steady state was reached, the product solution was collected for 30 s. The products can be obtained on a preparative scale with longer collection times.

Br-Li Exchange Reaction of *p*-Bromoanisole Followed by the Reaction with Methanol in Flow Microreactor Systems

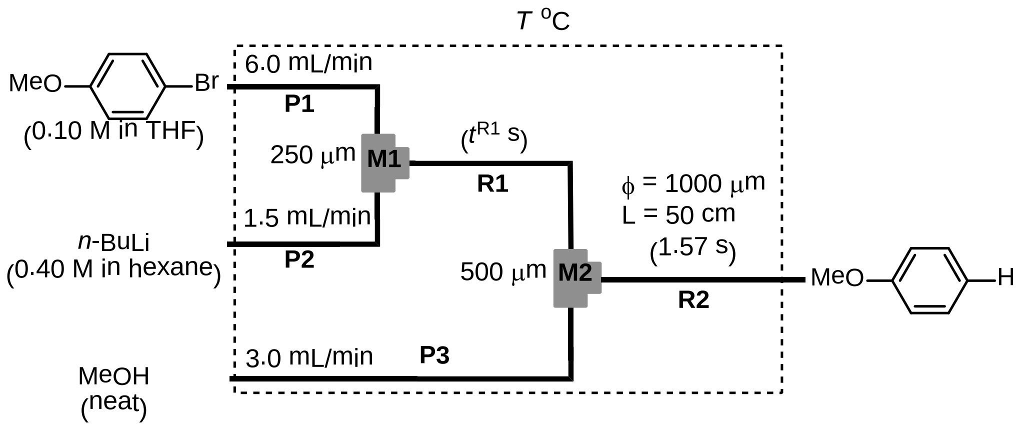

A flow microreactor system consisting of two T-shaped micromixers (**M1** and **M2**), two microtube reactors (**R1** and **R2**) and three tube pre-cooling units (**P1** (inner diameter ϕ = 1000 μm, length L = 100 cm), **P2** (ϕ = 1000 μm, L = 50 cm) and **P3** (ϕ = 1000 μm, L = 100 cm)) was used. A solution of *p*-bromoanisole (0.10 M in THF) (flow rate: 6.0 mL min^{-1}) and a solution of *n*-butyllithium (0.40 M in hexane) (flow rate: 1.5 mL min^{-1}) were introduced to **M1** (ϕ = 250 μm) by syringes pumps. The resulting solution was passed through **R1** (t^{R1} s) and was mixed with a solution of methanol (neat) (flow rate: 3.0 mL min^{-1}) in **M2** (ϕ = 500 μm). The resulting solution was passed through **R2** (ϕ = 1000 μm, L = 50 cm). The reactions were carried with varying residence time (t^{R1}: 0.22 ~ 6.3 s) and temperature (*T*: -78 ~ 24 °C). The yield of anisole was determined by GC. The results are summarized in Table S1.

Table S1: Br-Li Exchange Reaction of *p*-Bromoanisole Followed by the Reaction with Methanol in Flow Microreactor Systems

temperature (°C)	inner diameter of **R1** (μm)	length of **R1** (cm)	residence time in **R1**	yield (%)
24	1000	3.5	0.22	82
	1000	6.0	0.38	86
	1000	12.5	0.79	85
	1000	25	1.6	86
	1000	50	3.1	88
	1000	100	6.3	83
0	1000	3.5	0.22	83
	1000	6.0	0.38	84
	1000	12.5	0.79	84
	1000	25	1.6	84
	1000	50	3.1	85
	1000	100	6.3	88
-18	1000	3.5	0.22	77
	1000	6.0	0.38	83
	1000	12.5	0.79	85
	1000	25	1.6	85
	1000	50	3.1	86
	1000	100	6.3	86
-28	1000	3.5	0.22	72
	1000	6.0	0.38	87
	1000	12.5	0.79	90
	1000	25	1.6	90
	1000	50	3.1	88
	1000	100	6.3	89
-38	1000	3.5	0.22	65
	1000	6.0	0.38	73
	1000	12.5	0.79	86
	1000	25	1.6	84
	1000	50	3.1	87
	1000	100	6.3	85
-48	1000	3.5	0.22	66
	1000	6.0	0.38	67
	1000	12.5	0.79	87
	1000	25	1.6	88
	1000	50	3.1	88
	1000	100	6.3	90
-58	1000	3.5	0.22	45
	1000	6.0	0.38	45
	1000	12.5	0.79	77
	1000	25	1.6	84
	1000	50	3.1	92
	1000	100	6.3	90

-68	1000	3.5	0.22	29
	1000	6.0	0.38	44
	1000	12.5	0.79	63
	1000	25	1.6	84
	1000	50	3.1	85
	1000	100	6.3	85
-78	1000	3.5	0.22	12
	1000	6.0	0.38	17
	1000	12.5	0.79	62
	1000	25	1.6	67
	1000	50	3.1	84
	1000	100	6.3	83

Oxidative Homocoupling Reaction of *p*-Methoxyphenyllithium Using $FeCl_3$ in an Integrated Flow Microreactor System

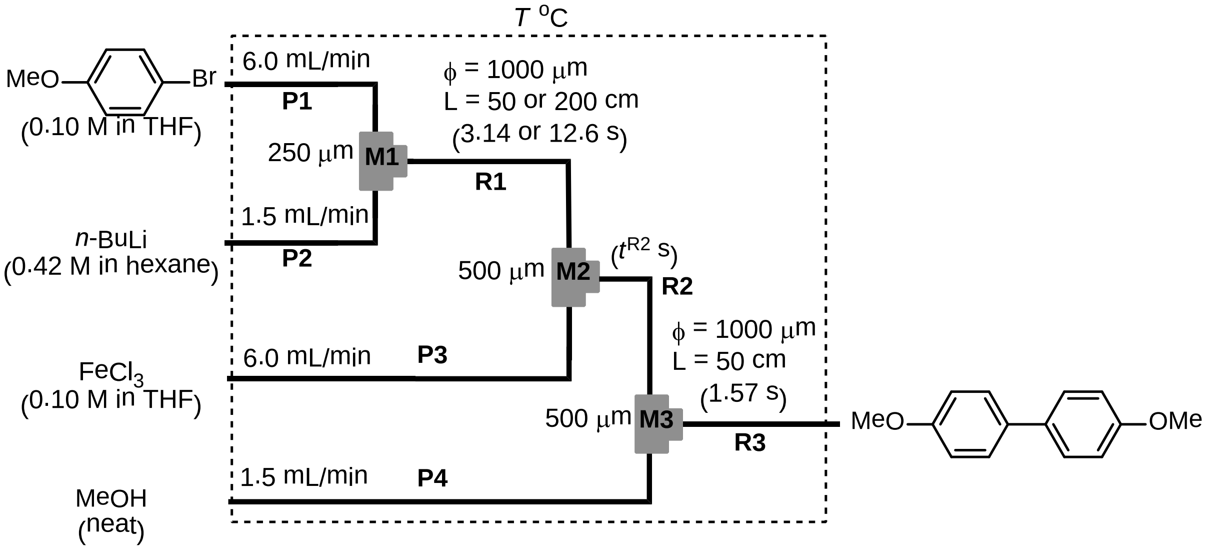

An integrated flow microreactor system consisting of three T-shaped micromixers (**M1**, **M2** and **M3**), three microtube reactors (**R1**, **R2** and **R3**) and four tube pre-cooling units (**P1** (inner diameter φ = 1000 μm, length L = 100 cm), **P2** (φ = 1000 μm, L = 50 cm) and **P3** (φ = 1000 μm, L = 100 cm), **P4** (φ = 1000 μm, L = 50 cm)) was used. A solution of *p*-bromoanisole (0.10 M in THF) (flow rate: 6.0 mL min^{-1}) and a solution of *n*-butyllithium (0.42 M in hexane) (flow rate: 1.5 mL min^{-1}) were introduced to **M1** (φ = 250 μm) by syringe pumps. The resulting solution was passed through **R1** (φ = 1000 μm, L = 50 cm (*T* = 24, 0 and -28 °C) or 200 cm (*T* = -48 and -78 °C)) and was mixed with a solution of $FeCl_3$ (0.10 M

in THF) (flow rate: 6.0 mL min^{-1}) in **M2** (ϕ = 500 μm). The resulting solution was passed through **R2** (t^{R2} s) and was mixed with methanol (flow rate: 1.5 mL min^{-1}) in **M3** (ϕ = 500 μm). The resulting solution was passed through **R3** (ϕ = 1000 μm, L = 50 cm). After a steady state was reached, the product solution was collected for 30 s. The reactions were carried with varying residence time (t^{R2}: 0.22 ~ 3.1 s) and temperature (*T*: -78 ~ 24 °C). The yield of 4,4'-dimethoxybiphenyl was determined by GC. The results are summarized in Table S2. In the case of t^{R2} = 3.1 s at 24 °C, sat. NH_4Cl solution (2 mL) was added and the organic materials were extracted with Et_2O (25 mL×3). The combined organic extracts were dried over Na_2SO_4 and concentrated. The crude product was purified by column chromatography to give 4,4'-dimethoxybiphenyl (21.7 mg, 68%) (Figure S1, S2).

Table S2: Oxidative Homocoupling Reaction of *p*-Methoxyphenyllithium Using $FeCl_3$ in an Integrated Flow Microreactor System

temperature (°C)	inner diameter of **R2** (μm)	length of **R2** (cm)	residence time in **R2** (s)	condition	yield (%)
24	1000	3.5	0.22	A	67
	1000	6.0	0.38		72
	1000	50	0.79		72
	1000	100	1.6		71
	1000	200	3.1		65
0	1000	3.5	0.22	A	61
	1000	6.0	0.38		72
	1000	50	0.79		73
	1000	100	1.6		73
	1000	200	3.1		69
-28	1000	3.5	0.22	A	53
	1000	6.0	0.38		63
	1000	50	0.79		66
	1000	100	1.6		71
	1000	200	3.1		69
-48	1000	3.5	0.22	B	45
	1000	6.0	0.38		59
	1000	50	0.79		67
	1000	100	1.6		74
	1000	200	3.1		71

-78	1000	3.5	0.22	B	21
	1000	6.0	0.38		22
	1000	50	0.79		23
	1000	100	1.6		35
	1000	200	3.1		51

Condition A: **R1** (ϕ = 1000 μm, L = 50 cm, 3.14 s), Condition B: **R1** (ϕ = 1000 μm, L = 200 cm, 12.6 s)

Typical Procedure for Oxidative Homocoupling Reaction of Various Aryllithiums Using $FeCl_3$ in an Integrated Flow Microreactor System

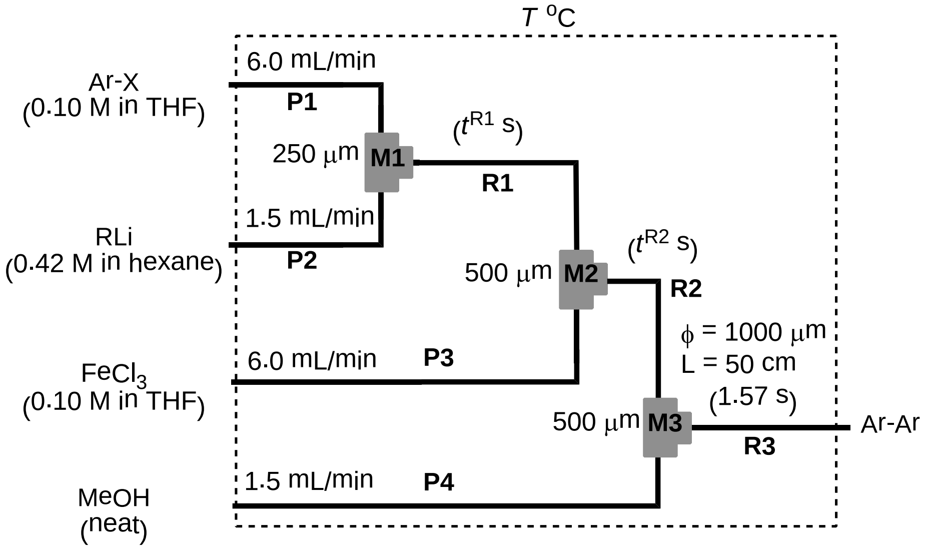

An integrated flow microreactor system consisting of three T-shaped micromixers (**M1**, **M2** and **M3**), three microtube reactors (**R1**, **R2** and **R3**) and four tube pre-cooling units (**P1** (inner diameter ϕ = 1000 μm, length L = 100 cm), **P2** (ϕ = 1000 μm, L = 50 cm) and **P3** (ϕ = 1000 μm, L = 100 cm), **P4** (ϕ = 1000 μm, L = 50 cm)) was used. A solution of aryl halides (ArX) (0.10 M in THF) (flow rate: 6.0 mL min^{-1}) and a solution of lithium reagents (0.42 M) (flow rate: 1.5 mL min^{-1}) were introduced to **M1** (ϕ = 250 μm) by syringe pumps. The resulting solution was passed through **R1** (t^{R1} s) and was mixed with a solution of $FeCl_3$ (0.10 M in THF) in **M2** (ϕ = 500 μm). The resulting solution was passed through **R2** (ϕ = 1000 μm) (t^{R2} s) was mixed with methanol (neat) (flow rate: 1.5 mL min^{-1}) in **M3** (ϕ = 500 μm). The resulting solution was passed through **R3** (ϕ = 1000 μm, L = 50 cm). After a steady state was reached, the product solution was collected for 30 s. The yield of homocoupling products was

determined by GC. The results are summarized in Table S3. The spectral data of all homocoupling products were identical to those of commercially available compounds.

Table S3: Typical Procedure for Oxidative Homocoupling Reaction of Various Aryllithium Using $FeCl_3$ in an Integrated Flow Microreactor System

Ar-X	lithium reagent	inner diameter of **R1** (μm)	length of **R1** (cm)	residence time in **R1** (s)	length of **R2** (cm)	residence time in **R2** (s)	temperature (°C)	yield (%)
m-bromoanisole	*n*-BuLi	1000	50	3.1	50	1.75	24	69
o-bromoanisole	*n*-BuLi	1000	50	3.1	200	6.98	0	76
p-bromobenzonitrile	*n*-BuLi	500	3.5	0.055	6	0.209	-28	75
m-bromobenzonitrile	*n*-BuLi	500	3.5	0.055	50	1.75	0	66
o -bromobenzonitrile	*n*-BuLi	500	3.5	0.055	50	1.75	24	76
p-iodonitrobenzene	PhLi	250	3.5	0.014	200	6.98	-48	53
o- iodonitrobenzene	PhLi	250	3.5	0.014	50	1.75	-48	63

3,3’-dimethoxybiphenyl: Obtained in 69% yield (GC ^{t}R 23.9 min) from *m*-bromoanisole.

2,2’-dimethoxybiphenyl: Obtained in 76% yield (GC ^{t}R 21.5 min) from *o*-bromoanisole.

1,1’-biphenyl-4,4’-dicarbonitrile: Obtained in 75% yield (GC ^{t}R 26.0 min) from *p*-bromobenzonitrile.

1,1’-biphenyl-3,3’-dicarbonitrile: Obtained in 66% yield (GC ^{t}R 25.6 min) from *m*-bromobenzonitrile.

1,1’-biphenyl-2,2’-dicarbonitrile: Obtained in 76% yield (GC ^{t}R 23.5 min) from *o*-bromobenzonitrile.

4,4’-dinitrobiphenyl: Obtained in 53% yield (GC ^{t}R 28.5 min) from *p*-iodonitrobenzene.

2,2’-dinitrobiphenyl: Obtained in 63% yield (GC ^{t}R 25.6 min) from *o*-iodonitrobenzene.

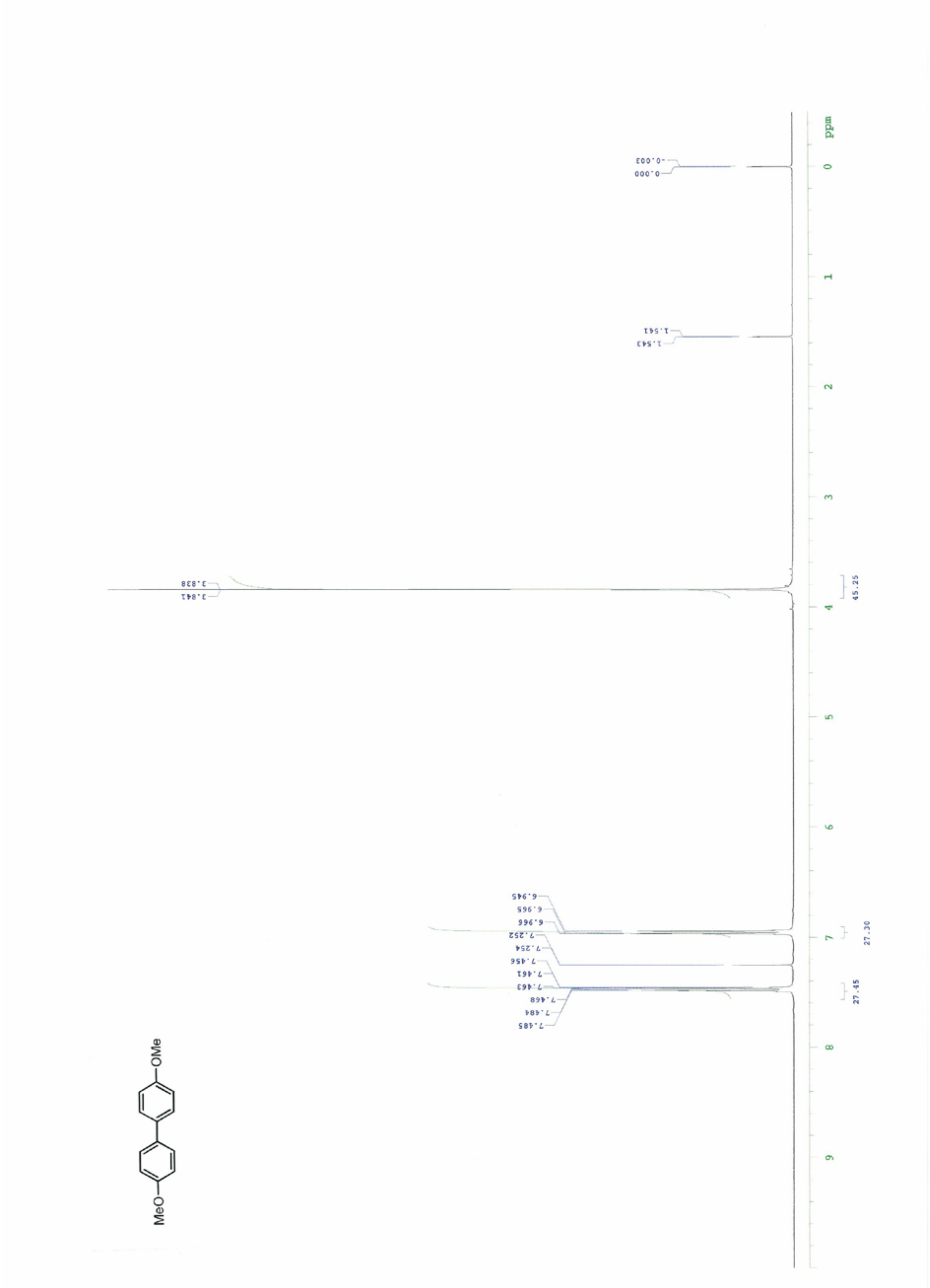

Figure S1: ^{1}H NMR spectrum of 4,4'-dimethoxybiphenyl.

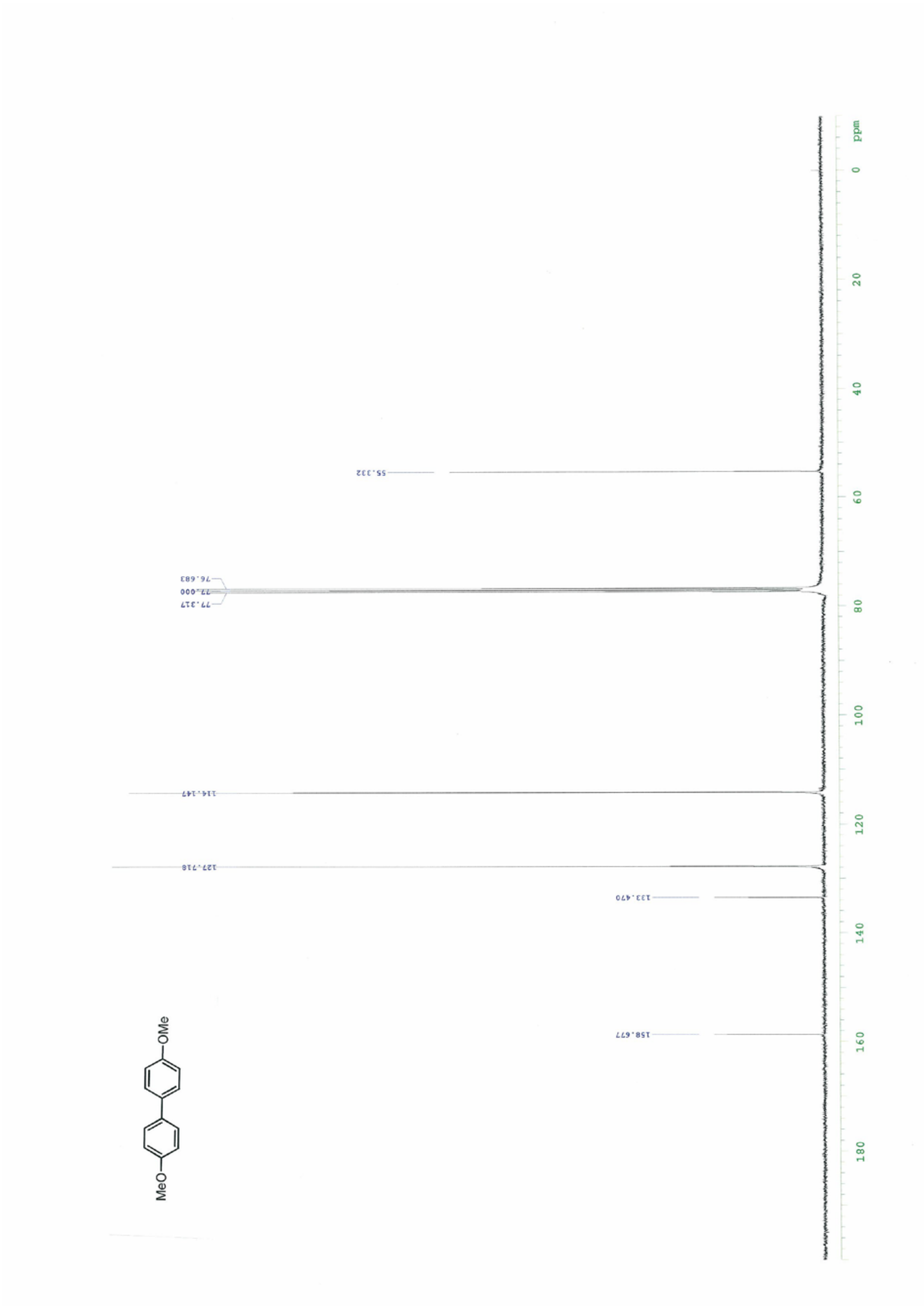

Figure S2: ^{13}C NMR spectrum of 4,4’-dimethoxybiphenyl.
